# Editorial: Gene editing to achieve Zero Hunger

**DOI:** 10.3389/fgeed.2025.1632120

**Published:** 2025-07-31

**Authors:** Shakeel Ahmad, Iqrar Ahmad Rana, Kevin M. Folta, Christian Damian Lorenzo, Sultan Habibullah Khan

**Affiliations:** ^1^ National Center for Genome Editing, Center for Advanced Studies in Agriculture and Food Security, University of Agriculture, Faisalabad, Pakistan; ^2^ Seed Center and Plant Genetic Resources Bank, Ministry of Environment, Water and Agriculture, Riyadh, Saudi Arabia; ^3^ Center of Agricultural Biochemistry and Biotechnology, University of Agriculture Faisalabad, Faisalabad, Pakistan; ^4^ Horticultural Sciences Department and the Graduate Program in Plant Molecular and Cellular Biology, University of Florida, Gainesville, FL, United States; ^5^ Center for Plant Systems Biology, VIB, Ghent, Belgium; ^6^ Department of Plant Biotechnology and Bioinformatics, Ghent University, Ghent, Belgium

**Keywords:** genome editing, site-directed nucleases (SDNs), food security and nutrition, SDG 2, NPBTs

Ensuring universal access to nutritious and healthy food remains a pressing global challenge. In 2015, the United Nations (UN) adopted 17 Sustainable Development Goals with Goal 2 (SDG 2): Zero Hunger, aiming to ensure global food security and to improve nutrition, sustainability in food production and resilience in agricultural practices, wellbeing and income of small-scale food producers, biodiversity conservation, and investment in agricultural research and gene banks by 2030 (United Nations, 2015). However, several persistent and emerging factors continue to threaten these targets, specifically food security and nutrition. These include a rapidly growing global human population, yield reductions imposed by climate change, and slow-paced plant breeding techniques. Furthermore, social and economic factors have further compromised the present scenario, along with the recent geopolitical conflicts (e.g., Russia-Ukraine, Palestine-Israel, civil war in Sudan, and other countries in East and Central Africa), the COVID-19 pandemic and even ravages derived from natural disasters (e.g., Hurricane Milton) (FAO, 2023). As of now, the global population has reached nearly eight billion and is projected to grow to 8.5 billion by 2030 and 10 billion by 2050. According to the Food and Agriculture Organization (FAO), current agricultural production falls short of meeting present demands and must double by 2030 to keep pace with this growth, highlighting a significant gap between food supply and demand (FAO et al., 2024). To meet this challenge, the rapid development of climate-resilient, high-yielding crop varieties is essential. This demands a transformative shift in breeding strategies, accelerating the process of developing cultivars that can withstand climate stress, deliver high productivity, and meet both regulatory standards and societal expectations.

While conventional breeding methods have substantially contributed to food security, indicators from the SDGs show that the current pace of progress is insufficient to meet expectations. In 2019, the FAO forewarned that continuing at the current pace in crop improvement would not suffice to eradicate hunger, which was also reported by Ahmad et al. (2021), and the same trends have been seen in the current report of 2023 (United Nations, 2023). This concern urges the world to move towards innovative and efficient biotechnology and breeding tools. Gene editing technologies (GETs), notably site-directed nucleases, have revolutionized crop improvement. Since its application in plants was demonstrated in 2013 (Shan et al., 2013), CRISPR/Cas has emerged as a robust and efficient tool for developing crops with enhanced yield, stress tolerance, and nutritional quality (Ahmad et al., 2025). The acceptance, deregulation, approval, and eventual commercialization of GETs-derived products can significantly accelerate progress towards Zero Hunger, aligning agricultural innovation with the 2030 SDGs. Hence, in light of the ongoing food security crisis, this Research Topic aims to present the current status, key advances, and future potential of GETs in achieving SDG 2. The Research Topic includes five articles: two original research articles and three review articles, each highlighting the potential of gene editing in the field of agriculture.

Recalcitrance of tropical maize lines to genetic transformation has always limited the application of advanced biotechnological tools for improving agronomically important tropical maize lines. In this regard, Jos Hernandes-Lopes et al. successfully enabled gene editing in transformation-recalcitrant agronomically important tropical maize lines using morphogenic regulator (MR)-assisted, agrobacterium-mediated transformation protocol. The *VIRESCENT YELLOW-LIKE (VYL)* gene, encoding a proteolytic subunit of the chloroplast Clp protease complex, was targeted using the CRISPR/Cas9 system and knockout mutants of three maize lines (CML360, CML444, and PCL1) were efficiently generated. The transformation efficiency remained up to 6.63% in these responsive lines, which was also confirmed by protoplast assays and inherited edits in subsequent generations. The findings of this research will potentially open the doors for gene editing in other recalcitrant tropical maize lines that could be important for food security and nutrition.

The legislation and regulatory processes regarding GETs, also referred to as *new plant breeding technologies* or *new genomic technologies*, are slow-paced in various countries, which are affecting countries’ agriculture and economies, thus lagging them behind the countries that are flexible in embracing the agricultural innovations. In this regard, Smyth et al. presented the findings of a survey conducted on Canada’s Plants with Novel Traits (PNTs) regulatory framework, established in the early 1990s. The plant breeders believe the PNTs framework has been outdated and is causing hindrance in developing new varieties using GETs. Thus, the authors have concluded that Canada’s PNTs regulations need to be updated and aligned with technological advancements to foster innovations in agriculture, pertinent to regional as well as global food security and nutrition.

Similarly, Kumari et al. have also summarized regulatory frameworks, guidelines, and legislations of various countries for nano-technology-based products, including the products developed through CRISPR/Cas-based gene editing system, in agriculture.

Patel et al. extensively reviewed the applications and methods of gene editing in various plant species. They summarized the mechanism of action of gene editing systems and their applications in agriculture for developing desirable traits in plants. This review also provides a comprehensive discussion on the regulation of gene-edited crops. Finally, it is concluded that the GETs are a cost-effective, efficient, robust, and innovative plant breeding tool that can help to meet the UN’s sustainable development goals of “zero hunger” and “good human health and wellbeing”.

Cotton is a major and economically important crop worldwide. It directly supports Target 2.3 of SDG 2, by contributing to the economic wellbeing and livelihood security of small-scale food producers, which is a key pillar of the Zero Hunger goal. In this regard, to cover the potential of CRISPR/Cas-based gene editing systems in cotton, Saleem et al. have extensively reviewed their applications in cotton. They have summarized the utilization of CRISPR/Cas9, CRISPR/nCas9, and CRISPR/Cas12a systems for targeting undesirable genes and improving the 4Fs–fiber, food, feed, and fuel–of cotton, highlighting how cotton improvement can contribute to achieving Zero Hunger by 2030.

In conclusion, GETs, particularly CRISPR-based systems, are evolving rapidly and demonstrating high efficiency, reliability, robustness, and effectiveness in generating new, transgene-free, desirable lines that may also bypass strict regulatory processes. As highlighted in various published reports, i.e., those by Patel et al. and Saleem et al., these systems have significantly improved a wide range of crops by targeting key traits, thereby contributing to the UN’s mission to achieve sustainable food security and nutrition, as well as to improve wellbeing and livelihoods of small-scale food producers globally. We hope that the articles featured in this Research Topic will further underscore the utility of GETs in crop improvement and pave the way for the deregulation of their products, enabling faster outcomes in support of the Zero Hunger goal.
